# Supplementary material for: The Eleanor ncRNAs activate the topological domain of the ESR1 locus to balance against apoptosis
Source: Nat Commun. 2019 Aug 22;10:3778. doi: 10.1038/s41467-019-11378-4 (PMC6706407; doi:10.1038/s41467-019-11378-4)
Supplement: Supplementary file 1 — Supplementary Information [file 41467_2019_11378_MOESM1_ESM.pdf]

Supplementary Information

The *Eleanor* ncRNAs activate the topological domain of the *ESR1* locus to balance against apoptosis

Abdalla and Yamamoto et al.,

Supplementary Figures

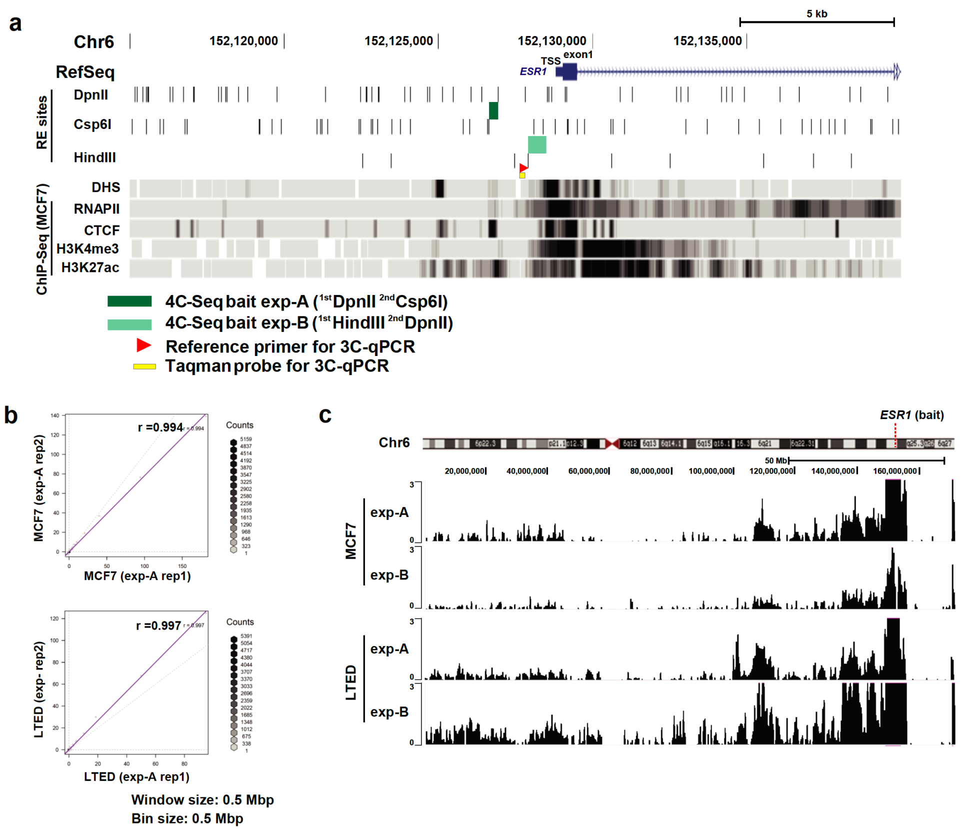

Supplementary Figure 1. Design and reproducibility of the 4C-Seq method.
a Top: Restriction enzyme (RE) sites around the TSS of the human *ESR1* gene (RefSeq NM_000125.3, transcript variant 1, chr6:152,128,814-152,424,408 [hg19]). The DNA region between DpnII and Csp6I (dark green bar) was used as a bait for the first 4C-Seq analysis (exp-A) and the region between HindIII and DpnII (light green bar) was used for the second 4C-Seq analysis (exp-B). Each 4C-Seq set was performed in duplicate (rep1 and rep2). For 3C-qPCR, the reference primer (red arrow) and Taqman probe (yellow bar) were designed at the HindIII restriction site proximal to the TSS of the *ESR1* gene. Bottom: alignment of selected ChIP-Seq data (UCSC genome browser) (**Supplementary Table 10**), showing that epigenetic marks typical of promoters were enriched at the TSS in MCF7 cells. They are DNase I hypersensitive sites (DHS), RNA polymerase II (RNAPII), CTCF, H3K4me3 and H3K27ac. **b** Hexbin plots show reproducibility of replicated 4C-Seq exp-A experiments (rep1 and 2) using MCF7 (r = 0.994) and LTED (r = 0.997) cells. **c** Two independent 4C-Seq profiles (exp-A and B) along the human chromosome 6 (hg19) showed a reproducible pattern of long-range chromosomal interactions with the *ESR1* promoter (bait) in MCF7 and LTED cells.

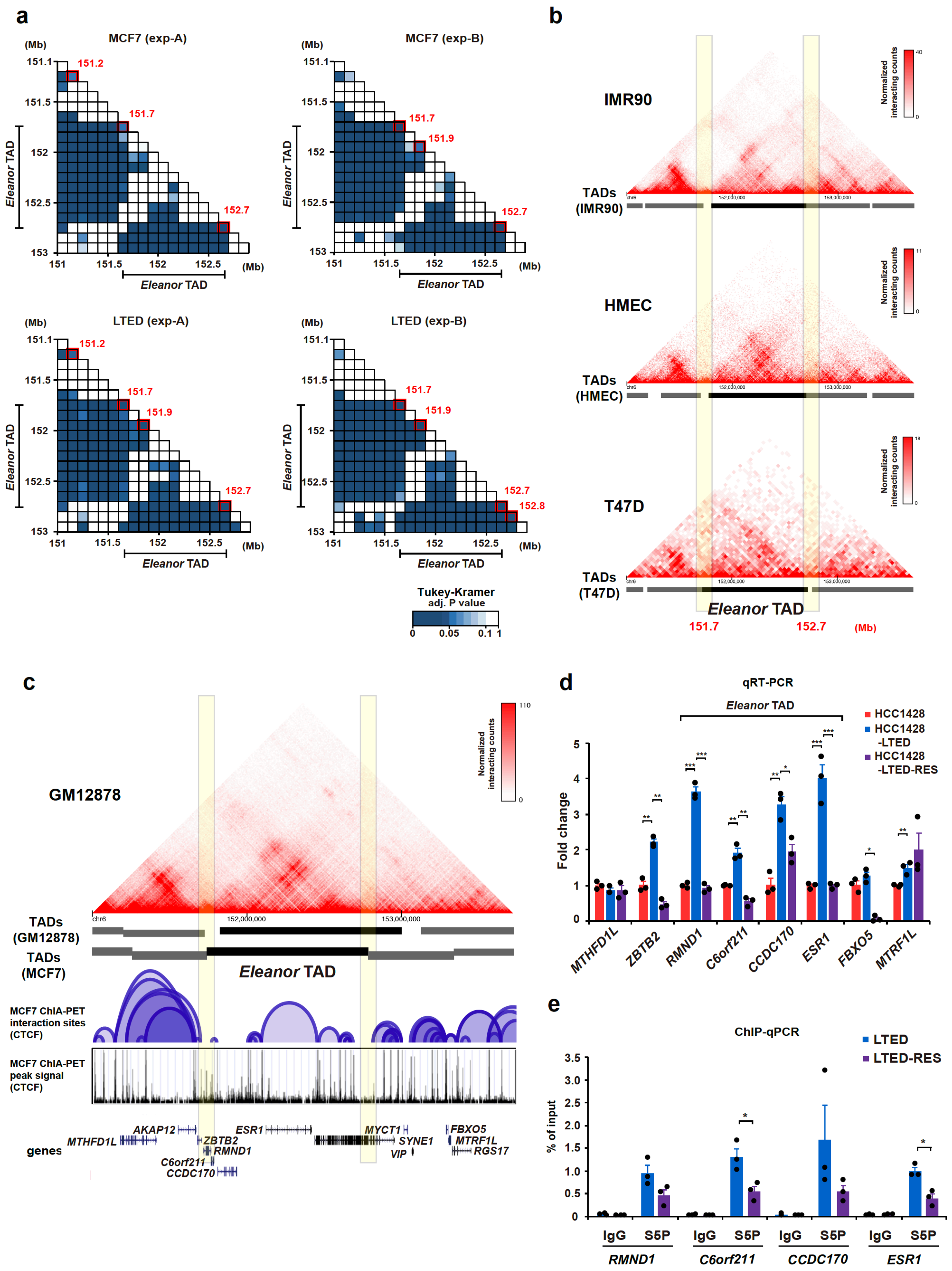

a
MCF7 (exp-A)
MCF7 (exp-B)
LTED (exp-A)
LTED (exp-B)
Eleanor TAD
Tukey-Kramer adj. P value
b
IMR90
HMEC
T47D
TADs (IMR90)
TADs (HMEC)
TADs (T47D)
Eleanor TAD
151.7
152.7
(Mb)
c
GM12878
TADs (GM12878)
TADs (MCF7)
MCF7 ChIA-PET interaction sites (CTCF)
MCF7 ChIA-PET peak signal (CTCF)
genes
d
qRT-PCR
HCC1428
HCC1428 -LTED
HCC1428 -LTED-RES
Fold change
e
ChIP-qPCR
LTED
LTED-RES
% of input

Supplementary Figure 2. Identification of the *Eleanor* TAD boundary.

a Quantification of the transitions of the 4C-Seq peaks. Triangular matrix showing the degrees of differences in 4C-Seq reads at every 100 kb throughout chr6: 151,000,000 – 153,000,000. Each box in the matrix represents the adjusted p value, from a multiple comparison using the Tukey-Kramer test. On the diagonal lines, the regions corresponding to the 4C-Seq peaks that are significantly different from the adjacent 100 kb bin are marked with red boxes, and they are considered to be the boundaries. **b** The locations of the *Eleanor* TADs in different cells. Hi-C profiles of the *Eleanor* surrounding region in IMR90 (human fetal lung cells, GSE63525[1]), HMEC (human mammary epithelial cells, GSE63525[1]) and T47D (human ER positive breast cancer cell line, GSE105697[2]) are shown. The black and grey bars represent TADs, and the yellow highlights delineate the positions of the *Eleanor* TAD boundaries. **c** *Eleanor* TAD and CTCF loops. The upper part is the contact matrix binned at a 10-kb resolution from GM12878 cells (lymphoblastoid cells, GSE63525[1]). The black and grey bars represent TADs, and the yellow highlights delineate the positions of the *Eleanor* TAD boundaries. The lower part shows the interactions and peaks of CTCF ChIA-PET in MCF7 cells (GSM970215[2]). **d** qRT-PCR analysis showing the expression levels of genes inside and outside the *Eleanor* TAD in another ER-positive human breast cancer cell line, HCC1428, its LTED cells (HCC1428-LTED), and the cells with resveratrol treatment (HCC1428-LTED-RES). The values in HCC1428 were set to 1. **e** ChIP-qPCR, showing that the resveratrol treatment suppressed RNA polymerase II binding to the TSSs of the genes in the *Eleanor* TAD. The value represents the percentage of input DNA, and IgG was used as a negative control. Data presented in **(d)** and **(e)** are representative of three independent experiments (mean ± s.e.m.). *P*-values were calculated using unpaired, two-tailed, Student's t-test (*$P < 0.05$, **$P < 0.01$, ***$P < 0.001$).

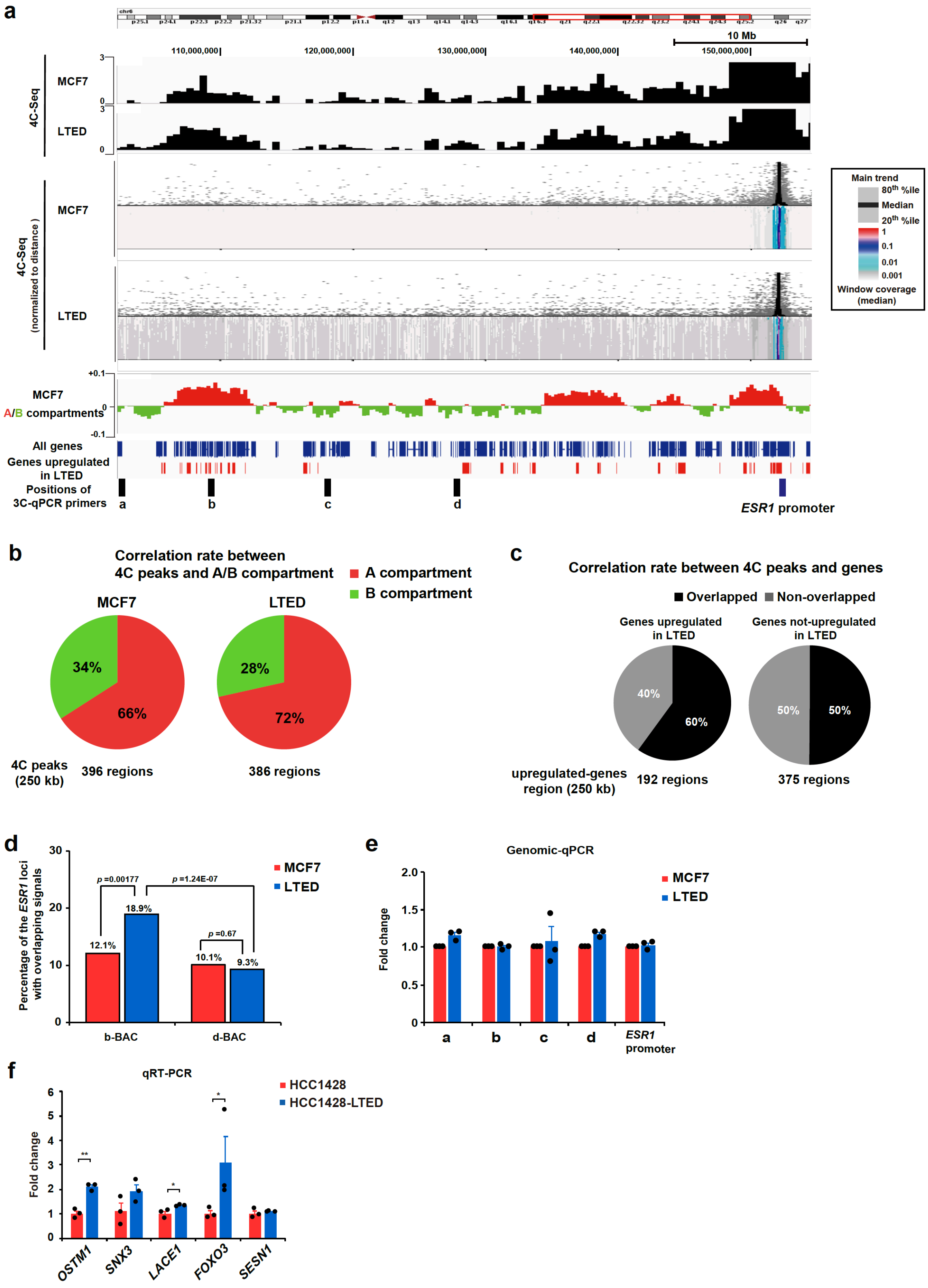

a
10 Mb
110,000,000
120,000,000
130,000,000
140,000,000
150,000,000
4C-Seq
MCF7
LTED
4C-Seq (normalized to distance)
Main trend
80th %ile
Median
20th %ile
Window coverage (median)
MCF7
A/B compartments
All genes
Genes upregulated in LTED
Positions of 3C-qPCR primers
a
b
c
d
ESR1 promoter
b
Correlation rate between 4C peaks and A/B compartment
A compartment
B compartment
MCF7
LTED
34%
66%
28%
72%
4C peaks (250 kb)
396 regions
386 regions
c
Correlation rate between 4C peaks and genes
Overlapped
Non-overlapped
Genes upregulated in LTED
Genes not-upregulated in LTED
40%
60%
50%
50%
upregulated-genes region (250 kb)
192 regions
375 regions
d
Percentage of the ESR1 loci with overlapping signals
MCF7
LTED
p =0.00177
p =1.24E-07
p =0.67
12.1%
18.9%
10.1%
9.3%
b-BAC
d-BAC
e
Genomic-qPCR
Fold change
MCF7
LTED
a
b
c
d
ESR1 promoter
f
qRT-PCR
Fold change
HCC1428
HCC1428-LTED
OSTM1
SNX3
LACE1
FOXO3
SESN1

Supplementary Figure 3. Long-range chromatin interaction of *ESR1*-6q21.
a An enlarged view of **Fig. 2a** (chr6:102,208,000-154,920,000). 4C-Seq data analyzed with pipeline described by van de Werken et al.[3] are also shown (normalized to distance). The normalized contact intensities and their median trend line are plotted in the top part, while the bottom part shows the medians for sliding windows (2-50 kb). **b** Correlation of the peaks of 4C-Seq and the A compartments. Overlapped regions in chromosome 6 were 66% and 72% in MCF7 and LTED cells, respectively. **c** Correlation of the 4C-Seq peaks and regions containing genes upregulated in LTED cells. The overlapping with the upregulated gene region was 60%, while that with the non-upregulated gene region was 50%. **d** Quantification of the DNA FISH analysis in **Fig. 2b**. The *ESR1* locus (visualized with *ESR1*p-BAC probe) with overlapping signals for sites b and d (visualized with b and d-BAC probes, respectively) were measured. *P*-values were calculated using two-tailed Fisher's exact test. **e** Copy number variation analysis was performed with genomic qPCR. Relative copy numbers of indicated genome sites were measured. The positions of a-d sites and the *ESR1* promoter are shown as thick blue bars in **Fig. 2a**. The genomic copy numbers of the tested sites were consistent in MCF7 and LTED cells. The values of MCF7 were set to 1. **f** Relative expression levels of genes at 6q21 in HCC1428 and HCC1428-LTED cells. *FOXO3* is activated in HCC1428-LTED cells. The values of HCC1428 were set to 1. Data presented in **(d), (e)** and **(f)** are representative of three independent experiments (mean ± s.e.m.). *P*-values were calculated using unpaired, two-tailed, Student's t-test (*$P < 0.05$, **$P < 0.01$, ***$P < 0.001$).

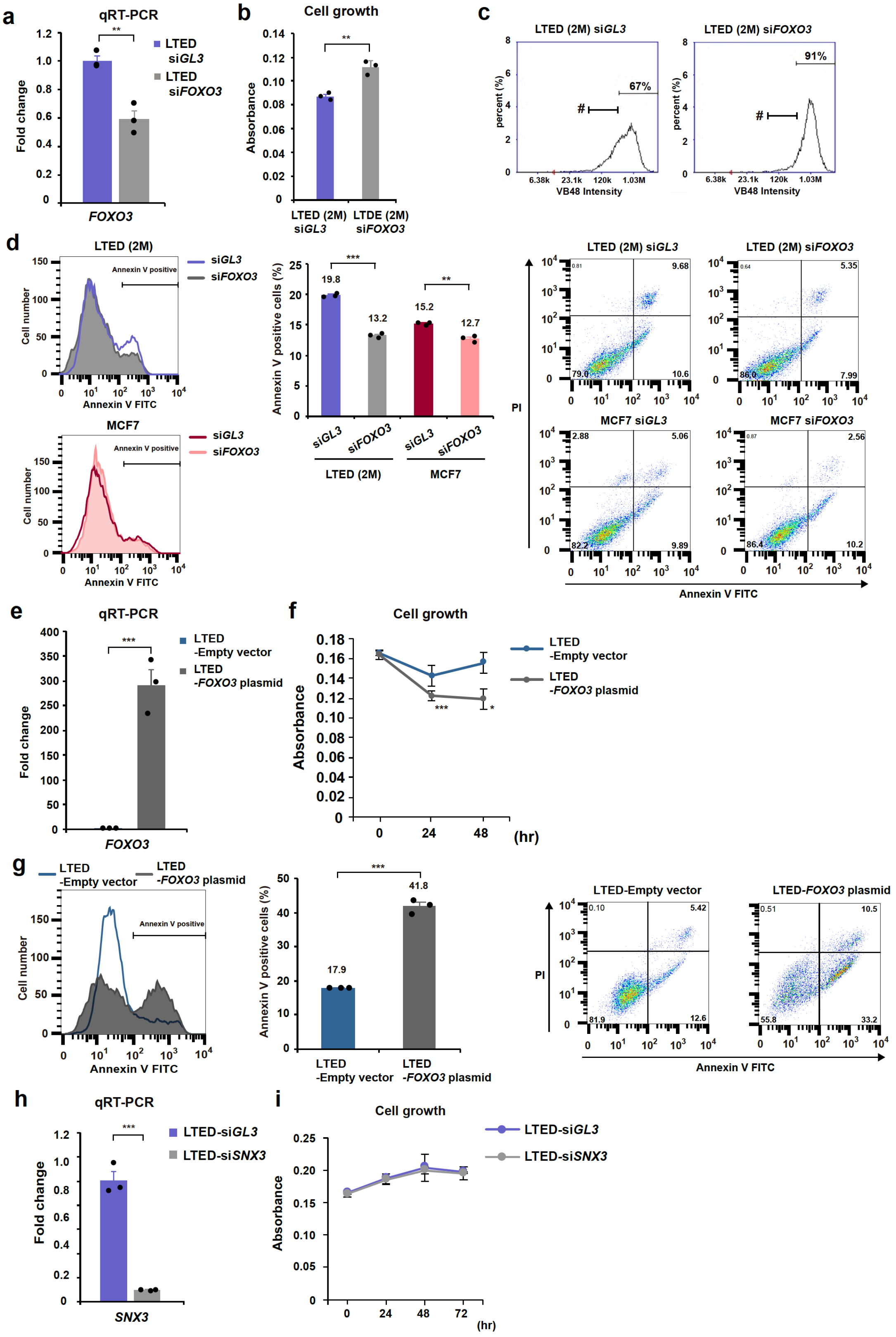
a
qRT-PCR
LTED siGL3
LTED siFOXO3
Fold change
FOXO3
b
Cell growth
Absorbance
LTED (2M) siGL3
LTDE (2M) siFOXO3
c
LTED (2M) siGL3
LTED (2M) siFOXO3
67%
91%
percent (%)
VB48 Intensity
d
LTED (2M)
MCF7
Annexin V positive
Cell number
Annexin V FITC
Annexin V positive cells (%)
19.8
13.2
15.2
12.7
PI
LTED (2M) siGL3
LTED (2M) siFOXO3
MCF7 siGL3
MCF7 siFOXO3
e
qRT-PCR
LTED -Empty vector
LTED -FOXO3 plasmid
f
Cell growth
(hr)
g
17.9
41.8
LTED-Empty vector
LTED-FOXO3 plasmid
h
qRT-PCR
LTED-siGL3
LTED-siSNX3
SNX3
i
Cell growth
LTED-siGL3
LTED-siSNX3

Supplementary Figure 4. Disruption of *FOXO3* restored cell vitality during estrogen deprivation.

a qRT-PCR analysis shows knockdown of *FOXO3* in LTED (2M) cells; the cells after two months of estrogen deprivation. Values of control siRNA (si*GL3*) were set to 1. **b** Cell proliferation assay showing the knockdown of *FOXO3* rescued cell death of LTED (2M) cells. The cell number was measured by the absorbance, using CCK-8. **c** A cell vitality assay with VB48 staining showed a decreased population of dying cells (denoted by bars with #) by knockdown of *FOXO3* in LTED (2M) cells. *FOXO3* knockdown increased the prevalence of VB48-negative, vital cells from 67% (si*GL3*) to 91% (si*FOXO3*). **d** FACS analysis of Annexin V-stained cells. The *FOXO3* knockdown decreased the apoptosis in LTED (2M) cells. A representative FACS profile is on the left. The quantified FACS analysis is in the middle. The plot data of Annexin V/PI-stained cells is on the right. **e** qRT-PCR analysis, showing *FOXO3* overexpression in LTED cells. The control (Empty vector) value was set to 1. **f** Overexpression of *FOXO3* suppressed LTED cell proliferation. Cell number was measured by the absorbance, using CCK-8. **g** FACS analysis of Annexin V-stained cells showing that *FOXO3* overexpression increased apoptosis in LTED cells. A representative FACS profile is on the left. Quantification of the FACS analysis is in the middle. A plot of the Annexin V/PI-stained cell data is on the right. **h** qRT-PCR analysis of LTED cells, showing that *SNX3* was knocked down with siRNA. Values of the control siRNA treatment (si*GL3*) were set to 1. **i** Knockdown of *SNX3* did not affect cell proliferation. Cell number was measured by the absorbance, using CCK-8. Data presented in **(a), (b), (d), (e), (f), (g), (h)** and **(i)** are representative of three independent experiments (mean ± s.e.m.). *P*-values were calculated using unpaired, two-tailed, Student's t-test (***P* < 0.01, ****P* < 0.001).

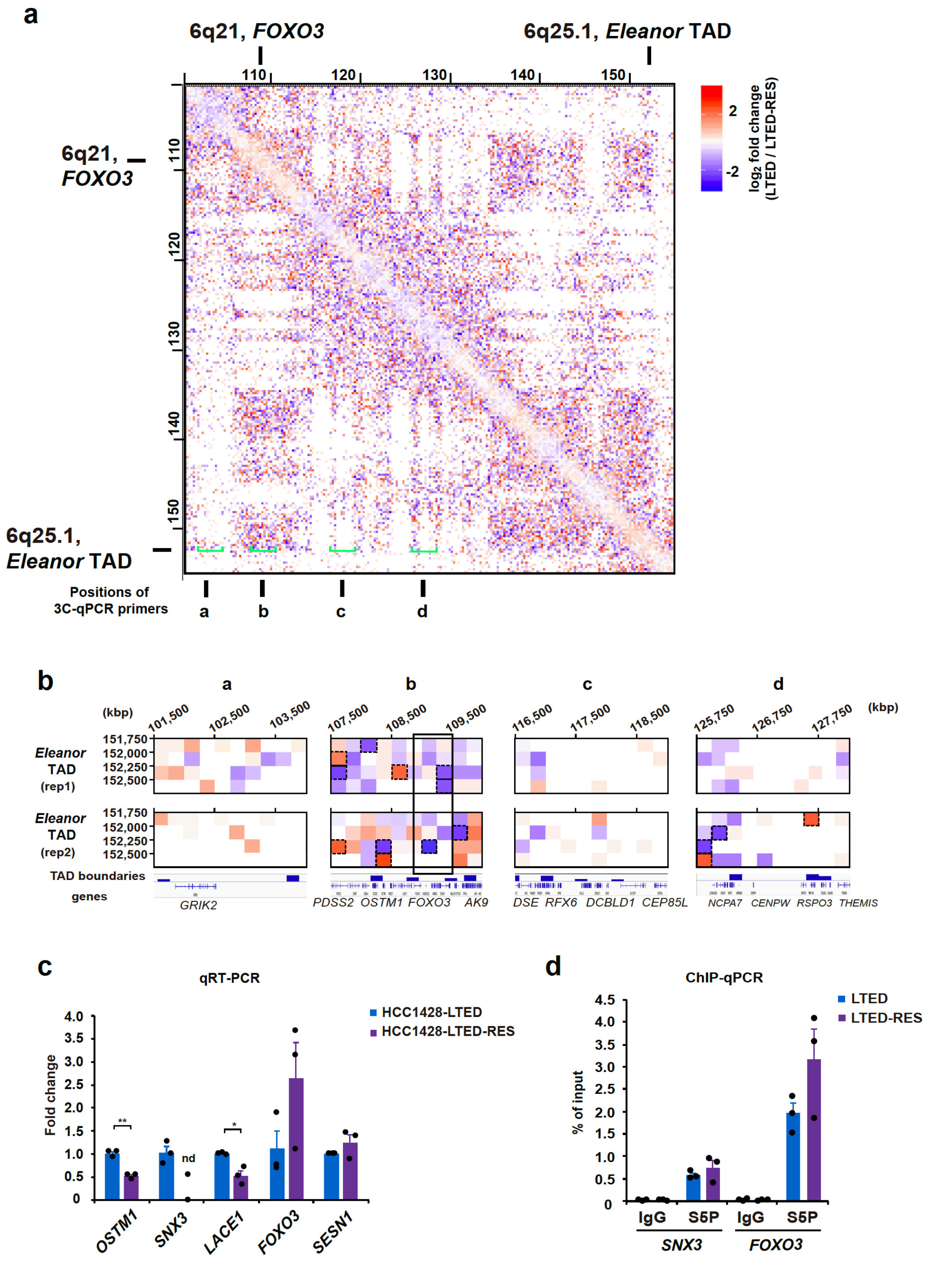

Supplementary Figure 5. Resveratrol treatment affected the local chromatin interactions.

a The $\log_2$ fold change in chromatin contacts between LTED and LTED-RES, derived from Hi-C in **Fig. 3a**. Gained (red) and reduced (blue) chromatin contacts are shown. **b** Enlarged

view of the areas marked with brackets in **Supplementary Fig. 5a**. The significantly different regions are enclosed by dotted boxes. **c** Relative expression levels of genes at 6q21 in HCC1428-LTED and HCC1428-LTED-RES cells. The values of HCC1428-LTED were set to 1. **d** ChIP-qPCR analysis showing that the resveratrol treatment had no impact on RNA polymerase II binding to the TSSs of *SNX3* and *FOXO3* in the 6q21 region. The value represents the percentage of input DNA, and IgG was used as a negative control. Data presented in **(c)** and **(d)** are representative of three independent experiments (mean ± s.e.m.). *P*-values were calculated using unpaired, two-tailed, Student's t-test (*$P < 0.05$, **$P < 0.01$, n.d. = not detected in at least one experiment).

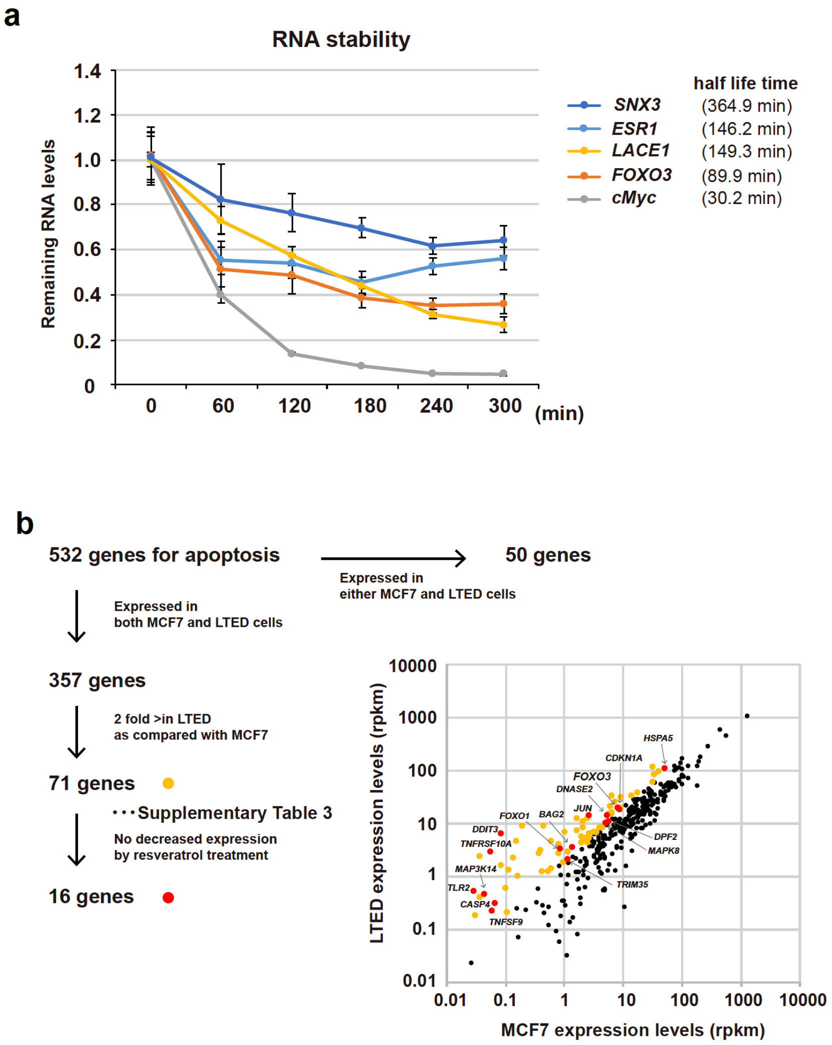

Supplementary Figure 6. RNA stability and regulation of apoptosis-related genes in LTED cells.

a RNA stability assay. LTED cells were treated with actinomycin D to inhibit new synthesis of RNA, and the levels of the remaining transcripts over time were measured by qRT-PCR. *cMyc* was used as an unstable RNA control. The half-life of each RNA is indicated on the right. **b** Apoptosis-related genes that were co-regulated with *FOXO3*. A scheme to extract genes that were upregulated in LTED cells and maintained at high levels in LTED-RES cells, similarly to the *FOXO3* gene. From 532 genes involved in apoptosis, 357 genes were extracted as being upregulated in LTED cells, according to our previous RNA-Seq[4]. They are plotted on the right graph. In total, 71 genes showed more than 2-fold higher expression in LTED cells (yellow dots), and each of them is listed in **Supplementary Table 3**. Among them, the high expression of 16 genes (red dots) was maintained with resveratrol treatment (listed in **Supplementary Table 3**).

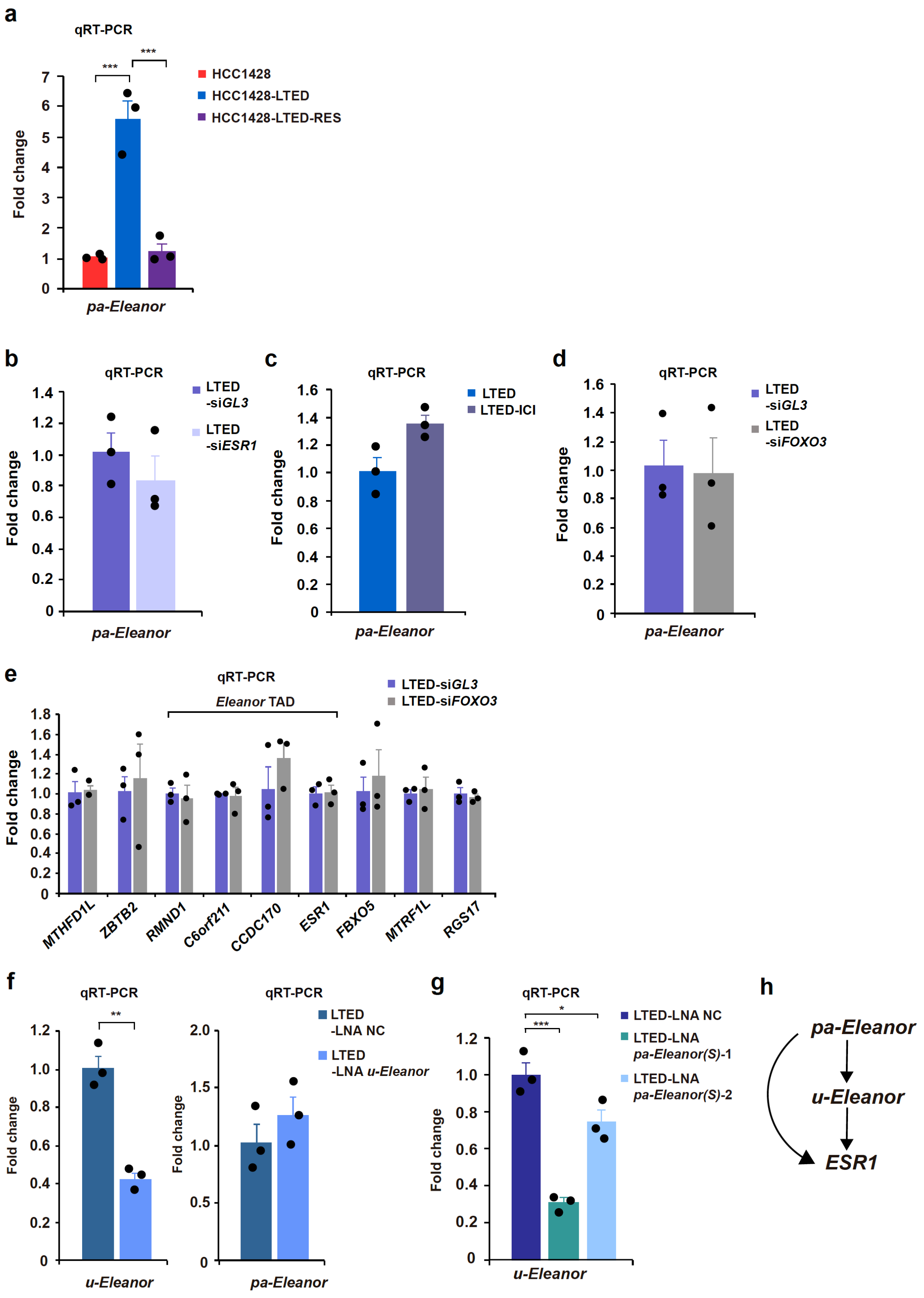
a
qRT-PCR
***
***
HCC1428
HCC1428-LTED
HCC1428-LTED-RES
Fold change
pa-Eleanor
b
qRT-PCR
LTED -siGL3
LTED -siESR1
Fold change
pa-Eleanor
c
qRT-PCR
LTED
LTED-ICI
Fold change
pa-Eleanor
d
qRT-PCR
LTED -siGL3
LTED -siFOXO3
Fold change
pa-Eleanor
e
qRT-PCR
Eleanor TAD
LTED-siGL3
LTED-siFOXO3
Fold change
MTHFD1L
ZBTB2
RMND1
C6orf211
CCDC170
ESR1
FBXO5
MTRF1L
RGS17
f
qRT-PCR
**
Fold change
u-Eleanor
qRT-PCR
LTED -LNA NC
LTED -LNA u-Eleanor
Fold change
pa-Eleanor
g
qRT-PCR
*
***
LTED-LNA NC
LTED-LNA pa-Eleanor(S)-1
LTED-LNA pa-Eleanor(S)-2
Fold change
u-Eleanor
h
pa-Eleanor
u-Eleanor
ESR1

Supplementary Figure 7. ***pa-Eleanor(S)*** **is transcribed independently of ER and FOXO3, and affects** ***u-Eleanor*** **transcription.**

a Expression levels of *pa-Eleanor(S)* in HCC1428 and their derivative cells. qRT-PCR results under the HCC1428 condition were set to 1. **b**, **c**, **d** Expression levels of *pa-Eleanor(S)* in LTED cells with *ESR1* mRNA knockdown **(b)**, ICI 182,780 treatment **(c)**, and *FOXO3* knockdown **(d)** showed no effect of each treatment. qRT-PCR results with control knockdown (si*GL3* in **b**, **d**), or no treatment (**c**) were set to 1. **e** Knockdown of *FOXO3* did not impact on transcriptional activities of genes around the *Eleanor* TAD in LTED cells. qRT-PCR analysis shows relative mRNA expression. Values with control siRNA (si*GL3*) were set to 1. **f** qRT-PCR analysis showing that the *u-Eleanor* knockdown did not impact the *pa-Eleanor* expression in LTED cells. The value of the LNA NC was set to 1. **g** qRT-PCR analysis showing that the *pa-Eleanor(S)* knockdown reduced the level of *u-Eleanor* expression. The value of the LNA NC was set to 1. **h** Schematic diagram, showing that *pa-Eleanor* either affects the transcription of *ESR1* through *u-Eleanor*, or directly affects the expression of *ESR1*. Data presented in **(a)** - **(g)** are representative of three independent experiments (mean ± s.e.m.). *P*-values were calculated using unpaired, two-tailed, Student's t-test (*$P < 0.05$, **$P < 0.01$, ***$P < 0.001$).

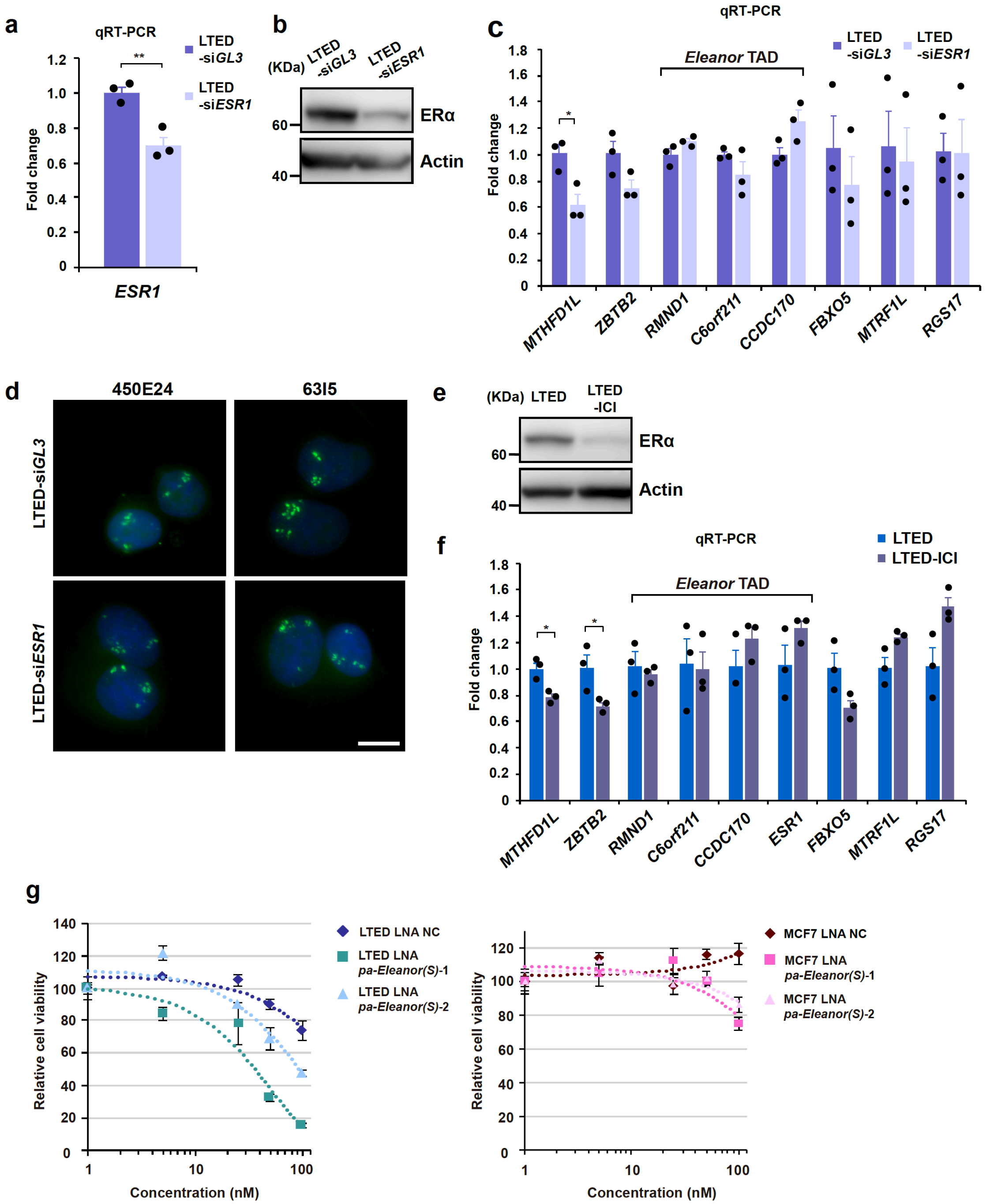

Supplementary Figure 8. ER plays a little to no role in *Eleanor* TAD regulation.
a qRT-PCR analysis of LTED cells, showing that the *ESR1* mRNA was knocked down with siRNA. The value of the control siRNA treatment (si*GL3*) was set to 1. **b** Immunoblot of LTED cells, showing that the amount of the ERα protein was reduced with si*ESR1*. Actin was used as a loading control. **c** The knockdown of the *ESR1* mRNA had little effect

on the transcriptional activities of genes at 6q25.1. Values of the control siRNA treatment (si*GL3*) were set to 1. **d** RNA FISH analysis shows that the *Eleanor* cloud did not change significantly after *ESR1* mRNA knockdown in LTED cells. Scale bar, 10 µm. **e** Immunoblot of LTED cells, showing that the amount of the ERα protein was reduced with the ICI 182,780 treatment. Actin was used as a loading control. **f** qRT-PCR analysis of LTED cells shows that degradation of ER with ICI 182,780 had little effect on the transcriptional activities of genes in the *Eleanor* TAD. Values of the control treatment were set to 1. **g** Reduction of cell viability by *pa-Eleanor(S)* knockdown. Relative cell proliferation of LTED (left) or MCF7 (right) was measured with a colorimetric assay 48 h after the indicated LNA treatment. Cell survival was inhibited in LTED cells by *pa-Eleanor(S)* knockdown in a dose-dependent manner. The value of 1 nM LNA GapmeR was set to 100. Data presented in **(a), (c), (f)** and **(g)** are representative of three independent experiments (mean ± s.e.m.). *P*-values were calculated using unpaired, two-tailed, Student's t-test (*$P < 0.05$, **$P < 0.01$).

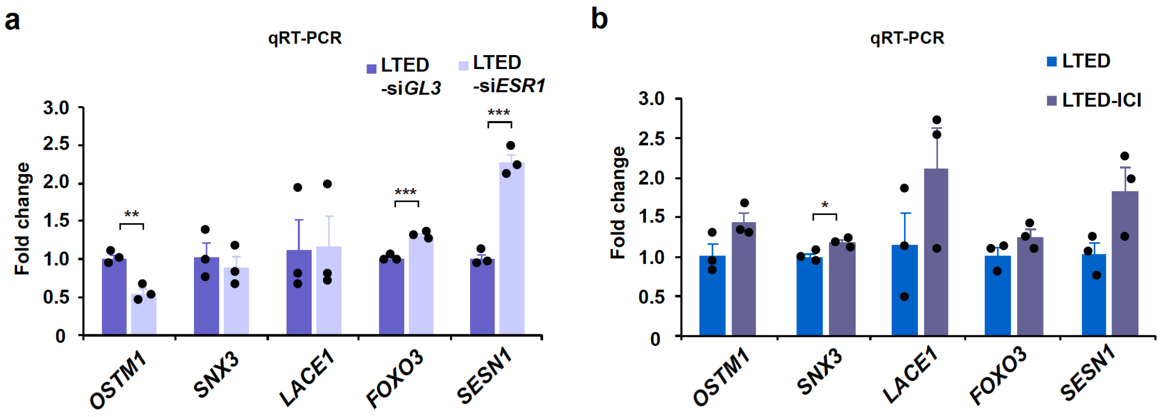

Supplementary Figure 9. ER plays little to no role in the transcriptional activities of genes at 6q21.

a, b qRT-PCR analysis of LTED cells shows that knockdown of *ESR1* mRNA **(a)** or degradation of ER with ICI 182,780 treatment **(b)** had little effect on the transcriptional activities of genes at 6q21. Values of the control RNAi (si*GL3* in **a**) or the control treatment (in **b**) were set to 1. Data presented in **(a)** and **(b)** are representative of three independent experiments (mean ± s.e.m.). *P*-values were calculated using unpaired, two-tailed, Student's t-test (*$P < 0.05$, **$P < 0.01$, ***$P < 0.001$).

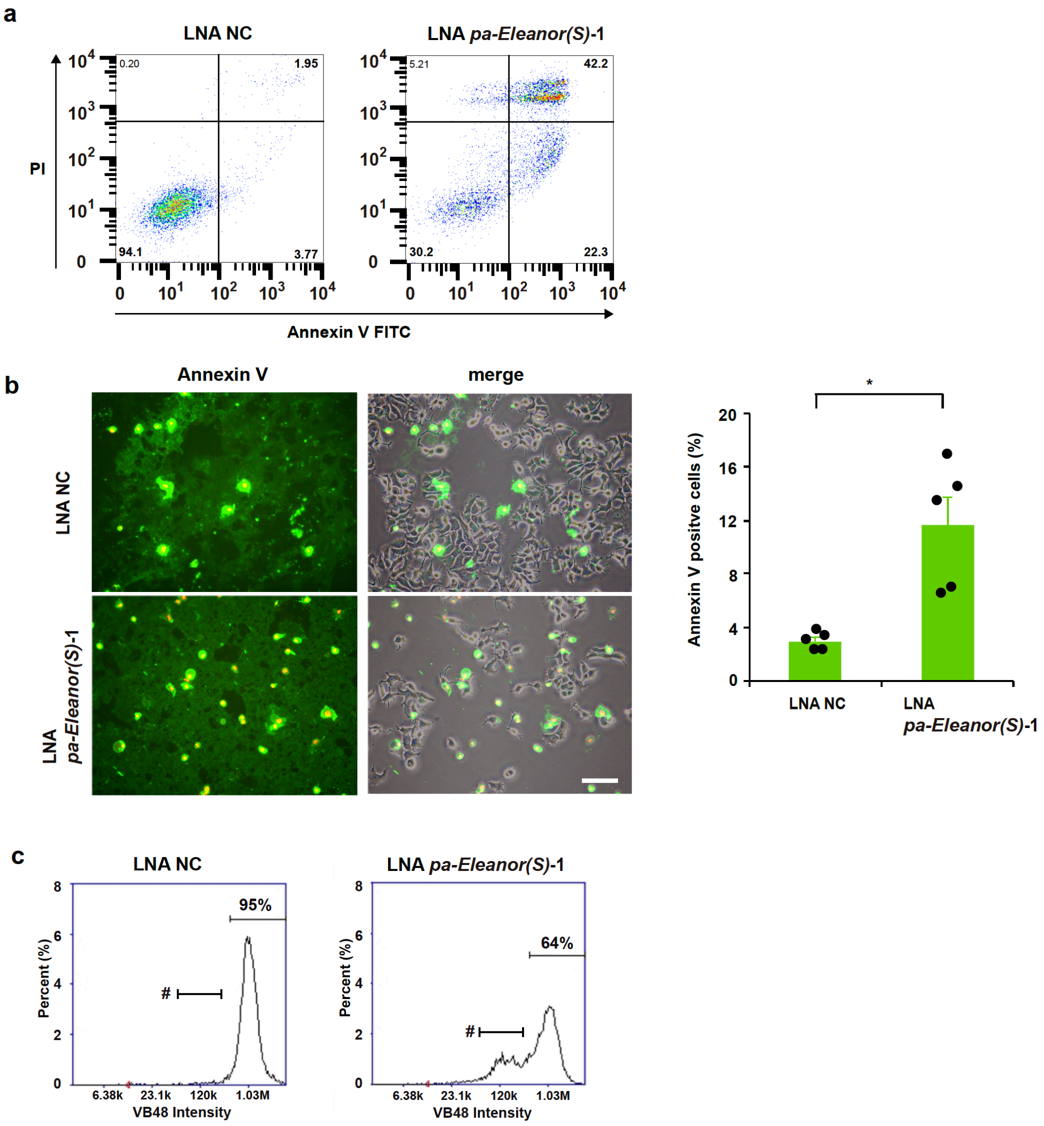

Supplementary Figure 10. Knockdown of *pa-Eleanor(S)* reduced viability of LTED cells.

a FACS plot data corresponding to **Fig. 6c**. **b** Microscopic analysis of Annexin V-stained LTED cells with *pa-Eleanor(S)* knockdown (left). Representative images show Annexin V signals (green, fluorescence) among total cells (gray, phase contrast). Scale bar, 100 µm. Ratio of number of Annexin V-positive cells over total cells was quantified (right). Cells in five optical fields (400 to 600 cells per field) were counted. Apoptotic cells increased upon knockdown of *pa-Eleanor(S)* in LTED cells. Data are representative of five independent experiments (mean ± s.e.m.). *P*-values were calculated using unpaired, two-tailed, Student's t-test (**P* < 0.05). **c** Vitality assay with VB48 staining shows that knockdown of

pa-Eleanor(S) in LTED cells increased a population of dying cells (denoted by bars with #).

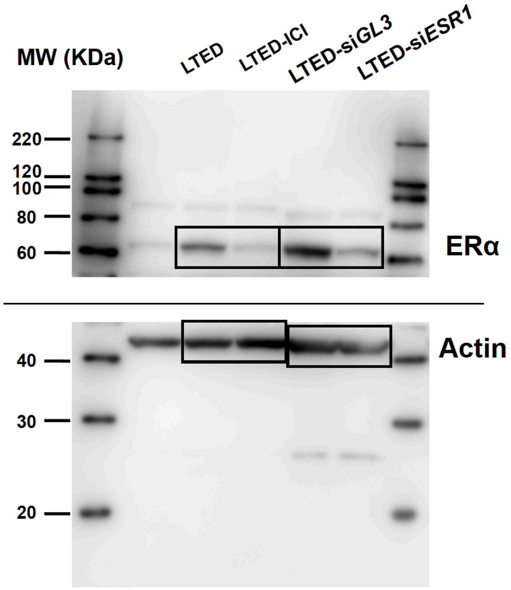

Supplementary Figure 11. Original immunoblot images.
Uncropped immunoblots for **Supplementary Fig. 8b** and **8e**. Separate blots are delineated with black lines.

Supplementary Tables

Supplementary Table 1. Expression levels of genes at 6q25.1 in MCF7, LTED, and LTED-RES cells.

	genes	MCF7 Expression values (RPKM)	LTED Expression values (RPKM)	LTED-RES Expression values (RPKM)
6q25.1 locus	***MTHFD1L***	**7.884686969**	**7.313863095**	**10.49561961**
	AKAP12	0.577163637	1.855117774	0.187807389
	ZBTB2	**8.726953582**	**7.396786185**	**3.847683898**
	RMND1	**38.56220229**	**71.79058937**	**31.373874**
	C6orf211	**92.41004688**	**119.9331546**	**41.81873099**
	CCDC170	**13.94896244**	**25.71870484**	**28.55330658**
	ESR1	**38.14162994**	**108.479172**	**23.80005886**
	SYNE1	0.605214684	1.116396996	0.225296831
	MYCT1	0	0	0.01748651
	VIP	0	0.392126746	0.235306166
	FBXO5	**22.33742688**	**9.575210193**	**4.104186825**
	MTRF1L	**11.93910162**	**7.828176498**	**9.377851331**
	RGS17	**3.508778501**	**4.53609663**	**3.436420015**

Supplementary Table 2. Expression levels of genes at 6q21 in MCF7, LTED, and LTED-RES cells.

	genes	MCF7 Expression values (RPKM)	LTED Expression values (RPKM)	LTED-RES Expression values (RPKM)
6q21 locus	***OSTM1***	**5.079195051**	**2.9895385**	**10.68027664**
	NR2E1	0	0	0.049014812
	SNX3	**84.60785254**	**111.7946926**	**46.29965677**
	LACE1	**3.791980403**	**0.617833654**	**2.212716109**
	FOXO3	**7.724661895**	**21.12612774**	**22.00814491**
	LINC00222	-	-	-

	ARMC2	0	0.659710454	0.499233977
	SESN1	**4.826175252**	**4.511779459**	**9.429206971**

Supplementary Table 3. Candidate genes for apoptosis in LTED cells.

genes	MCF7 RNA-Seq - RPKM	LTED RNA-Seq - RPKM	LTED-plus-RES RNA-Seq - RPKM	region	MCF7 Compart ment	LTED Compart ment
**DDIT3**	**0.07829435**	**6.65683419**	**33.7197217**	12q13.3	A	A
**TNFRSF10A**	**0.05270329**	**2.97254481**	**12.9921274**	8p21.3	A	B
**TLR2**	**0.02710278**	**0.54757164**	**2.15927314**	4q31.3	B	B
**MAP3K14**	**0.04129427**	**0.46928802**	**1.20391511**	17q21.31	A	A
**JUN**	**2.52572921**	**14.2992328**	**43.0073432**	1p32.1	B	B
**CASP4**	**0.06504284**	**0.32852368**	**0.60080591**	11q22.3	B	B
**TNFSF9**	**0.05539384**	**0.23315644**	**0.70355635**	19p13.3	A	B
**FOXO1**	**0.83583112**	**3.36922845**	**4.05519122**	13q14.11	A	A
**BAG2**	**1.34774597**	**3.74401777**	**5.49841298**	6p12.1	A	A
**DNASE2**	**5.28213952**	**14.4610408**	**16.281526**	19p13.13	A	A
**FOXO3**	**7.7246619**	**21.1261277**	**22.0081449**	**6q21**	A	A
**CDKN1A**	**8.23420122**	**19.2726124**	**51.7803145**	6p21.2	A	A
**HSPA5**	**49.5072359**	**113.136962**	**389.275618**	9q33.3	A	A
**MAPK8**	**4.83142944**	**10.6893382**	**13.3384258**	10q11.22	A	A
**DPF2**	**5.46822623**	**11.4466912**	**12.4171502**	11q13.1	A	A
**TRIM35**	**1.09097172**	**2.2225154**	**3.9427799**	8p21.2	A	B
DAPK2	0.03509541	2.45213507	1.37776135	15q22.31	A	A
BCL2	0.18111779	9.57026196	7.06198793	18q21.33	A	A
MAP2K5	0.14349397	4.8922044	0.68344301	15q23	A	A
TNFRSF6B	0.07985346	1.6805432	0.3227056	20q13.33	A	A
DUSP22	0.43593197	9.06948655	6.29177864	6p25.3	A	A
CDKN2D	0.1296286	2.2915854	1.64641086	19p13.2	A	A
CARD14	0.10545478	1.36710825	0.25569973	17q25.3	A	A
ARHGEF6	0.0349889	0.41235787	0.20199706	Xq26.3	A	A
ANGPTL4	0.38772779	3.30474449	1.90265729	19p13.2	A	A
BCL3	0.34636011	2.91570639	1.28545551	19q13.32	A	A
MAPK12	0.57841405	4.82490276	0.57678566	22q13.33	A	A
NFKB2	1.57023677	12.7082797	9.33561851	10q24.32	A	A
TGFB1	0.96423056	7.19944707	3.26739419	19q13.2	A	A
APLP1	0.15058081	1.04577803	0.69546349	19q13.12	A	A
DUSP7	0.02851028	0.19200271	0.13167585	3p21.2	A	A
DUSP6	0.09811782	0.63324298	0.03776342	12q21.33	A	A
BAG3	5.99087324	34.0340393	5.90246238	10q26.11	A	A
NOL3	1.94785109	10.9861741	7.67490692	16q22.1	A	A

BCL2A1	0.78244538	4.19904124	2.25859698	15q25.1	A	A
BCL2L11	2.43493929	13.0110078	8.52868508	2q13	A	A
ERCC2	1.52447519	7.44327861	3.46761834	19q13.32	A	A
MYO18A	1.93389016	8.82587691	7.632713	17q11.2	A	A
CLU	30.636088	124.578667	74.1354055	8p21.1	A	B
DAP	8.62448081	32.9858626	8.61673554	5p15.2	A	A
IGF2R	7.01038914	26.0739485	18.6254128	6q25.3	B	B
BOK	0.78011399	2.89549646	1.35112033	2q37.3	A	A
VEGFB	5.85723005	21.250604	12.6210217	11q13.1	A	A
NME3	1.81914693	5.89582286	0.78766899	16p13.3	A	A
TNFSF13	0.40523176	1.29629246	0.32752633	17p13.1	A	A
BCL6	1.06732439	3.04704678	1.31274202	3q27.3	A	A
MADD	6.02693136	16.5283992	5.91257856	11p11.2	A	A
BAG1	5.59623704	15.3357703	14.9029495	9p13.3	A	A
SON	13.0684447	34.9777228	15.494287	21q22.11	A	A
BAK1	2.48135622	6.62055856	3.1078624	6p21.31	A	A
DNASE1	2.11374538	5.53863208	1.06561597	16p13.3	A	A
STAT1	33.2658583	86.4504717	52.595802	2q32.2	A	A
BNIP3	38.239085	98.4938543	77.9952505	10q26.3	A	A
PPM1F	0.49806383	1.25783067	0.89343083	22q11.22	A	A
TIAL1	6.17522642	15.2604233	6.29288194	10q26.11	A	A
SEMA4D	0.58382485	1.44012632	1.03467215	9q22.2	A	A
TNFAIP1	2.95409593	7.11387305	5.36797762	17q11.2	A	A
TNFRSF12A	8.74986778	21.0450211	16.0821725	16p13.3	A	A
CRADD	1.86168039	4.44036545	2.95564674	12q22	A	A
APAF1	2.41126142	5.61443051	3.54679975	12q23.1	B	A
IKBKB	3.17036223	7.3111562	3.80239531	8p11.21	A	B
EP300	3.84250924	8.78236799	3.66483205	22q13.2	A	A
MAP2K1	17.2263928	39.210855	14.2278727	15q22.31	A	A
CBL	2.50560065	5.59469551	3.20133037	11q23.3	A	A
MX1	0.10233648	0.21537058	0.13293143	21q22.3	A	A
PTK2	30.6514181	63.8751799	39.8150989	8q24.3	A	A
RBBP6	5.56663472	11.5118946	6.23666257	16p12.1	A	A
SMNDC1	3.40921941	6.96435655	4.51374317	10q25.2	A	A
IRF1	0.93424507	1.90061208	1.79785336	5q31.1	A	A
CLN3	4.35424138	8.77488779	1.75202825	16p12.1	A	A
ESPL1	2.31168721	4.65401103	0.12863397	12q13.13	A	A

Supplementary Table 4. Primer sequences for qRT-PCR.

6q25.1 genes	sequence
MTHFD1L(ex4-ex5)_F	5’-AGCAGTGAAGCCGAGATTA-3'
MTHFD1L(ex5)_R	5’-GCATTGAGGACTTTGTTGCT-3'
ZBTB2(ex1)_F	5’-AGCGAGAGTTTGGTTTCCTGT-3'

ZBTB2(ex1-2)_R	5'-ACACTCACTGGTCTGATGGAC-3'
RMND1(ex3)_F	5'-AGCAACGGCAGATGAGTATC-3'
RMND1(ex4)_R	5'-CCAGGATCACCTTCTTTTGC-3'
C6orf211(ex3)_F	5'-CCGTGGTTGTTGGTAGAATG-3'
C6orf211(ex3-4)_R	5'-AATCGATTGGTGGACTCTGG-3'
CCDC170(ex1-2)_F	5'-AGCGCCCGAGGAAACTTAC-3'
CCDC170(ex2)_R	5'-AGCATTTTGAGCCACATTCC-3'
ESR1(ex1-ex2)_F	5'-CAGGCCAAATTCAGATAATCG-3'
ESR1(ex2)_R	5'-TCCTTGGCAGATTCCATAGC-3'
FBXO5(ex2)_F	5'-AATGGGCCTAGAATGTGTAGA-3'
FBXO5(ex3)_R	5'-GATCTTCTTCCAAGTTGTGCT-3'
MTRF1L(ex5-ex6)_F	5'-CCAACAGGTGTTGTTTCTGA-3'
MTRF1L(ex6)_R	5'-GCACGTAACTTTGTCATAGCC-3'
RGS17(ex3-ex4)_F	5'-GAATGCCAAAACCCCACTG-3'
RGS17(ex4)_R	5'-TTCACAAGCAAGCCAGAAAA-3'
**6q21 genes**	**sequence**
OSTM1(ex4-ex5)_F	5'-GATGCAATGAACATCACTCG-3'
OSTM1(ex5)_R	5'-CAGCAATTACAGGCACTGTGT-3'
SNX3(ex1)_F	5'-ATGACACCGCTTCTCCTCAC-3'
SNX3(ex1)_R	5'-AAACGGCTCACTAGCTGGAA-3'
LACE1(ex12-13)_F	5'-CTGAGCCAGGATTCAGCAG-3'
LACE1(ex13)_R	5'-TCAGTCTGCATTTCCGTGAG-3'
FOXO3(ex2)_F	5'-GGGTCCAGAATGAGGGAACT-3'
FOXO3(ex2)_R	5'-CCACGGCTCTTGGTATACTTG-3'
*SESN1*_F	5'-TTCGTGTCCAGGACTATTGC-3'
*SESN1*_R	5'-CATCTTTGTGCATTGCCATT-3'
GAPDH	**sequence**
GAPDH(ex8)_F	5'-ACACCCACTCCTCCACCTTT-3'
GAPDH(ex8-9)_R	5'-TAGCCAAATTCGTTGTCATACC-3'
cMyc	**sequence**
c-Myc-RTqPCR-S	5'-GCCACGTCTCCACACATCAG-3'
c-Myc-RTqPCR-AS	5'-TCTTGGCAGCAGGATAGTCCTT-3'

Supplementary Table 5. BAC clones for FISH and 3C-qPCR.

**Clone ID**	**regions**	position
RP-11-108N8	chr6: 151,720,298-151,895,171	*Eleanor* TAD

RP3-404G5	chr6: 151,959,800-152,079,536	*Eleanor* TAD
RP3-443C4	chr6: 152,079,437-152,146,782	ESR1p-BAC
RP11-450E24	chr6: 152,083,345-152,291,757	*Eleanor* TAD
RP1-63I5	chr6: 152,282,470-152,370,174	*Eleanor* TAD
RP1-130E4	chr6: 152,370,075-152,478,967	*Eleanor* TAD
RP11-438N24	chr6: 104,028,541-104,191,294	Chr6_a
RP11-787I22	chr6: 108,998,482-108,999,125	Chr6_b
RP4-747C14	chr6: 116,837,719-116,982,546	Chr6_c
RP11-515J24	chr6: 124,449,075-124,631,584	Chr6_d
RP11-369N23	chr12: 6,463,209-6,662,155	GAPDH

Supplementary Table 6. Primer sequences for 3C-qPCR and genomic DNA qPCR.

**Name in figures**	**sequence**
*ESR1*p_Reference(Taqman)	5'-TGAACCGAGAAGATCGAGTTG-3'
a	5'-GCCCAGGTAAAATAGGGAACA-3'
b	5'-ATGTACGTGATGTTGCCTCTGT-3'
c	5'-GTTCAGGAGTTTTGCCTGAGAT-3'
d	5'-TCCCTAGGTTTCACCAGTCAAT-3'
*ESR1*p-Taqman probe	5'-FAM-CATTGTCGGTCTGGTCCAGCTAACA"-TAMRA-3"
**Loading control**	**sequence**
*ESR1*_4C_L-control_F	5'-TCCTAGCAGGGAGATGAGGA-3'
*ESR1*_4C_L-control_R	5'-GCAAATGGCACAAAGAATCC-3'
**Digestion control at reference**	**sequence**
*ESR1*_4C_control_F	5'-TGCATATCCTAGCCCAAGTG-3'
*ESR1*_4C_control_R	5'-AACATAACCTCAGGTCACGAAC-3'
**3C normalization control primer**	**sequence**
*GAPDH*_C_1	5'-CATAGTGGGGTGGTGAATACC-3'
*GAPDH*_C_2	5'-CCCCAGGTTTACATGTTCCA-3'
**BAC quantification**	**sequence**
BACquant1_F	5'-caactcaatcgacagctgga-3'
BACquant1_R	5'-acggatttttccgtcagatg-3'
BACquant2_F	5'-caacccggtaagacacgact-3'
BACquant2_R	5'-gcctacatacctcgctctgc-3'
**genomic DNA**	**sequence**
Chr6_a_F	5'-TGCCTCACTAATCTAACATTGC-3'

Chr6_a_R	5'-ATAGAGAAAGTGGTTGGCATAG-3'
Chr6_b_F	5'-ATGTACGTGATGTTGCCTCTGT-3'
Chr6_b_R	5'-AAGTGTGCATTCTGCCTCCT-3'
Chr6_c_F	5'-GTTCAGGAGTTTTGCCTGAGAT-3'
Chr6_c_R	5'-TTGGGAATACCCTTCTTCCA-3'
Chr6_d_F	5'-TCCCTAGGTTTCACCAGTCAAT-3'
Chr6_d_R	5'-TCAGCAAGGGTGCATAAGTG-3'

Supplementary Table 7. Primer sequences for ChIP-qPCR.

**6q25.1 genes**	**sequence**
*RMND1*_promoter F	5’-ACTACTGCAGCCAACCCATC-3'
*RMND1*_promoter R	5’-CCCGAGTTTCTCCCGCTAAG-3'
*C6orf211*_promoter F	5’-CTCTGTTTCTGCGGCGATTG-3'
*C6orf211*_promoter R	5’-AGACTAACCCCACGTCCTGT-3'
*CCDC170*_promoter F	5’-TGGACTGCACCAGCCATATC-3'
*CCDC170*_promoter R	5’-TCCTGGGTCGTCTATCCCCA-3'
*ESR1*_promoter F	5’-TAAGCCAATGTCAGGGCAAG-3'
*ESR1*_promoter R	5’-TCCCGAGCTCATATGCATTAC-3'
**6q21 genes**	**sequence**
*SNX3*_promoter F	5’-GGGGAGAAACGGCTCACTAG-3'
*SNX3*_promoter R	5’-CATGACACCGCTTCTCCTCA-3'
*FOXO3*_promoter F	5’-AGAAGAGCCGAAGACAGCAC-3'
*FOXO3*_promoter R	5’-GGTGTGCGTGCGTTTGTTTA-3'

Supplementary Table 8. Primer sequences for primer walking assay.

**Primer name**	**sequence**
*ESR1*paRNA_F for (e), (f)	5’-TGCCCTATCTCGGTTACAGTG-3'
*ESR1*paRNA_R for (e)	5’-TACAAAGGTGCTGGAGGACG-3'
ESR1-exon1_F for (g)	5’-CCTCTAACCTCGGGCTGTGC-3'
ESR1-exon1_R for (f), (g)	5’-TGGGCTCGTTCTCCAGGTAGT-3'

Supplementary Table 9. Antisense LNA and siRNA sequences.

**Antisense LNA**	**sequence**
LNA NC (Negative Control A)	5’-AACACGTCTATACGC-3’
LNA *pa-Eleanor(S)*-1	5’-GTGCCAGACTCCGATA-3’
LNA *pa-Eleanor(S)*-2	5’-TCCAGGCACAACTCGA-3’

LNA *u-Eleanor(S)*	5’-GCGTGACTGAGGAATA-3’
**siRNA**	**sequence**
siRNA-*GL3*	5'-CUUACGCUGAGUACUUCGA-3'
siRNA-*ESR1*	5'-GGGCTCTACTTCATCGCAT-3'

Supplementary Table 10. ChIP-Seq and ChIA-PET data used in this study.

Data	File Name
RNA Pol ll ChIP-Seq (Crawford, lyer-UT Austin)	wgEncodeOpenChromChipMcf7Pol2Sig.bigWig
CTCF ChIP-Seq (Crawford, lyer-UT Austin)	wgEncodeOpenChromChipMcf7CtcfSig.bigWig
H3K4me3 ChIP Seq (Stamatoyannopoulous, UW)	wgEncodeUwHistoneMcf7H3k4me3StdRawRep 1.bigWig
H3K27ac ChIP-Seq (Snyder, Farnham-USC)	wgEncodeSydhHistoneMcf7H3k27acUcdSig.big Wig
DHS (DNase I hypersensitive sites) (Crawford - Duke University)	wgEncodeOpenChromDnaseMcf7Sig.bigWig
CTCF ChIA-PET Interaction and Signal (ENCODE/GIS-Ruan)	wgEncodeGisChiaPetMcf7CtcfInteractionsRep1. bed.gz wgEncodeGisChiaPetMcf7CtcfSigRep1.bigWig

Supplementary References

1 Rao, S. S. *et al.* A 3D map of the human genome at kilobase resolution reveals principles of chromatin looping. *Cell* **159**, 1665-1680 (2014).

2 Consortium, E. P. An integrated encyclopedia of DNA elements in the human genome. *Nature* **489**, 57-74 (2012).

3 van de Werken, H. J. G. *et al.* Robust 4C-seq data analysis to screen for regulatory DNA interactions. *Nature Methods* **9**, 969 (2012).

4 Tomita, S. *et al.* A cluster of noncoding RNAs activates the ESR1 locus during breast cancer adaptation. *Nat Commun* **6**, 6966 (2015).
