# Supplementary material for: The Eleanor ncRNAs activate the topological domain of the ESR1 locus to balance against apoptosis
Source: Nat Commun. 2019 Aug 22;10:3778. doi: 10.1038/s41467-019-11378-4 (PMC6706407; doi:10.1038/s41467-019-11378-4)
Supplement: Supplementary file 3 — Reporting Summary [file 41467_2019_11378_MOESM3_ESM.pdf]

nature research

Corresponding author(s): Noriko Saitoh, Mitsuyoshi Nakao

Last updated by author(s): Jun 14, 2019

Reporting Summary

Nature Research wishes to improve the reproducibility of the work that we publish. This form provides structure for consistency and transparency in reporting. For further information on Nature Research policies, see Authors & Referees and the Editorial Policy Checklist.

Statistics

For all statistical analyses, confirm that the following items are present in the figure legend, table legend, main text, or Methods section.

n/a	Confirmed	
☐	☒	The exact sample size (*n*) for each experimental group/condition, given as a discrete number and unit of measurement
☐	☒	A statement on whether measurements were taken from distinct samples or whether the same sample was measured repeatedly
☐	☒	The statistical test(s) used AND whether they are one- or two-sided<br>*Only common tests should be described solely by name; describe more complex techniques in the Methods section.*
☒	☐	A description of all covariates tested
☐	☒	A description of any assumptions or corrections, such as tests of normality and adjustment for multiple comparisons
☐	☒	A full description of the statistical parameters including central tendency (e.g. means) or other basic estimates (e.g. regression coefficient) AND variation (e.g. standard deviation) or associated estimates of uncertainty (e.g. confidence intervals)
☐	☒	For null hypothesis testing, the test statistic (e.g. *F*, *t*, *r*) with confidence intervals, effect sizes, degrees of freedom and *P* value noted<br>*Give P values as exact values whenever suitable.*
☒	☐	For Bayesian analysis, information on the choice of priors and Markov chain Monte Carlo settings
☒	☐	For hierarchical and complex designs, identification of the appropriate level for tests and full reporting of outcomes
☒	☐	Estimates of effect sizes (e.g. Cohen's *d*, Pearson's *r*), indicating how they were calculated

Our web collection on statistics for biologists contains articles on many of the points above.

Software and code

Policy information about availability of computer code

Data collection	Data collected from public repository (e.g. ENCODE) was directly retrieved from the associated website without the use of custom software.<br>Flow cytomentry data were acquired using BD FACSCanto™ II or BD FACSCalibur™.<br>qPCR data were acquired using ABI Prism 7300 and Step One Plus system.<br>Sequencing was performed using Illumina HiSeq1500 and Hiseq X Ten.<br>Images (e.g. FISH) were obtained using an IX-71 microscope (Olympus) equipped with image acquisition software (Lumina Vision Version 2.4; Mitani Corporation), a confocal laser-scanning microscope (LSM 780, Carl Zeiss) with image acquisition LSM software (Carl Zeiss), or a microscope CKX53 (Olympus) with CellSens standard program (v 1.16).<br>Immunoblotting were acquired with Amersham Imager 600 (GE Healthcare)<br>Cell vitality data was assessed using Automated Cell Analyzer (NucleoCounter NC-250, Chemometec).<br>Cell proliferation data was measured by a microplate reader (BIO-RAD).
Data analysis	For 4C-seq analyses, Bowtie 2 (v2.1.0), SAMtools (v0.1.18), Integrative Genomics Viewer (v2.3.68) , deepTools (v3.13), bedtools (v2.27.1), Homer (v4.10) and 4C pipeline developed by de Laat and colleagues were used.<br>For RNA-seq analyses, Tophat (v1.4.1) and Cufflinks (v1.3.0) as previously described in Tomita et al., Nat commun 2015.<br>For Hi-C analyses, Juicer pipeline, juicer tools (v1.9.9), UCSC Genome Browser Utilities, R package (HiCcompare (v1.4.0), ggplot2 (v3.1.0)) and Hi-C browser were used. For image analysis, Cellomics CellInsight with HCS studio or Image J (v1.49) were used. Details are included in the Method section were used.

For manuscripts utilizing custom algorithms or software that are central to the research but not yet described in published literature, software must be made available to editors/reviewers. We strongly encourage code deposition in a community repository (e.g. GitHub). See the Nature Research guidelines for submitting code & software for further information.

Data

Policy information about availability of data

All manuscripts must include a data availability statement. This statement should provide the following information, where applicable:

- Accession codes, unique identifiers, or web links for publicly available datasets
- A list of figures that have associated raw data
- A description of any restrictions on data availability

For RNA-Seq datasets in MCF7, LTED and LTED-RES cells: DRA001006 (Figs. 1b, 2a, 4a, b, S3a, c, S6b)
For 4C-Seq datasets in MCF7 and LTED cells: DRA006154 (Figs. 1b, 2a, S1b, c, S2a, 3a, b, c)
For Hi-C datasets in LTED and LTED-RES cells: DRA007945 (Figs. 3a, S5a, b)

Field-specific reporting

Please select the one below that is the best fit for your research. If you are not sure, read the appropriate sections before making your selection.

☒ Life sciences ☐ Behavioural & social sciences ☐ Ecological, evolutionary & environmental sciences

For a reference copy of the document with all sections, see nature.com/documents/nr-reporting-summary-flat.pdf

Life sciences study design

All studies must disclose on these points even when the disclosure is negative.

Sample size	Sample size was not pre-determined, we used sample sizes commonly used and accepted for the type of experiments. We used two biological replicates per conditions for 4C-Seq and Hi-C.
Data exclusions	No data were excluded from the analysis, except 3C-qPCR and 4C-Swq. For 3C-qPCR, we excluded Ct values that were clearly outlier in technical replicates. For 4C-Seq, according to the method, the self-ligated read was excluded.
Replication	Results presented in this study have been confirmed on independent datasets.<br>The replication number for each experiment is reported in each figure legend and individual data.
Randomization	This was not relevant to our study.
Blinding	This was not relevant to our study.

Reporting for specific materials, systems and methods

We require information from authors about some types of materials, experimental systems and methods used in many studies. Here, indicate whether each material, system or method listed is relevant to your study. If you are not sure if a list item applies to your research, read the appropriate section before selecting a response.

Materials & experimental systems

n/a	Involved in the study	
☐	☒	Antibodies
☐	☒	Eukaryotic cell lines
☒	☐	Palaeontology
☒	☐	Animals and other organisms
☒	☐	Human research participants
☒	☐	Clinical data

Methods

n/a	Involved in the study	
☒	☐	ChIP-seq
☐	☒	Flow cytometry
☒	☐	MRI-based neuroimaging

Antibodies

Antibodies used	Anti-Phospho RNA Polymerase II CTD (Ser5) mAb: MBL International Corporation, MABI0603; 2ug for ChIP<br>ERα: Santa Cruz Biotechnology, sc-543; 1:1000 dilution for immunoblotting<br>Actin: Sigma, A2103; 1:1000 dilution for immunoblotting
Validation	The commercial antibody used in this study was described on the manufacturer's website.

Eukaryotic cell lines

Policy information about cell lines

Cell line source(s)	MCF10A cells (purchased from ATCC), MCF7 cells (purchased from ATCC) and LTED cells were established by culturing MCF7 cells in estrogen deprivation culture medium<br>HCC1428 cells (purchased from ATCC) and HCC-1428LTED cells were established by culturing HCC1428 cells in estrogen deprivation culture medium
Authentication	All cells were not authenticated
Mycoplasma contamination	All cell lines were tested negative for mycoplasma contamination
Commonly misidentified lines<br>(See ICLAC register)	cell line used is not in the ICLAC database

Flow Cytometry

Plots

Confirm that:

☒ The axis labels state the marker and fluorochrome used (e.g. CD4-FITC).

☒ The axis scales are clearly visible. Include numbers along axes only for bottom left plot of group (a 'group' is an analysis of identical markers).

☒ All plots are contour plots with outliers or pseudocolor plots.

☒ A numerical value for number of cells or percentage (with statistics) is provided.

Methodology

Sample preparation	Cells were transfected with Antisense LNA GapmeR Negative Control A, LNA pa-Eleanor(S)-1 (TAKARA), siRNA-GL3 or siRNA-FOXO3 (CST, #6303) using RNAiMAX (Invitrogen). Cells were acquired in the indicated days.
Instrument	The number of fluorescent cells were monitored by FACS (BD FACSCanto™ II and BD FACSCalibur™ , Becton Dickinson).
Software	The data were analyzed using FlowJo (v10.4.1).
Cell population abundance	All cells were considered in the analyses
Gating strategy	In the initial gating FSC/SCC we only excluded debris or doublet cells that in general accounted, all single cells were considered in the analyses.

☒ Tick this box to confirm that a figure exemplifying the gating strategy is provided in the Supplementary Information.
